# Accuracy of CT cerebral perfusion in predicting infarct in the emergency department: lesion characterization on CT perfusion based on commercially available software

**DOI:** 10.1007/s10140-012-1102-8

**Published:** 2013-01-16

**Authors:** Chang Y. Ho, Sajjad Hussain, Tariq Alam, Iftikhar Ahmad, Isaac C. Wu, Darren P. O’Neill

**Affiliations:** 1 MRI Department, Riley Hospital for Children, 702 Barnhill Drive, Indianapolis, IN 46202 USA; 2 Department of Radiology, Indiana University School of Medicine, 550 N University Boulevard, Indianapolis, IN 46202 USA

**Keywords:** CT perfusion, Stroke, Diagnostic accuracy, CT perfusion software

## Abstract

This study aims to assess the diagnostic accuracy of a single vendor commercially available CT perfusion (CTP) software in predicting stroke. A retrospective analysis on patients presenting with stroke-like symptoms within 6 h with CTP and diffusion-weighted imaging (DWI) was performed. Lesion maps, which overlays areas of computer-detected abnormally elevated mean transit time (MTT) and decreased cerebral blood volume (CBV), were assessed from a commercially available software package and compared to qualitative interpretation of color maps. Using DWI as the gold standard, parameters of diagnostic accuracy were calculated. Point biserial correlation was performed to assess for relationship of lesion size to a true positive result. Sixty-five patients (41 females and 24 males, age range 22–92 years, mean 57) were included in the study. Twenty-two (34 %) had infarcts on DWI. Sensitivity (83 vs. 70 %), specificity (21 vs. 69 %), negative predictive value (77 vs. 84 %), and positive predictive value (29 vs. 50 %) for lesion maps were contrasted to qualitative interpretation of perfusion color maps, respectively. By using the lesion maps to exclude lesions detected qualitatively on color maps, specificity improved (80 %). Point biserial correlation for computer-generated lesions (*R*_pb_ = 0.46, *p* < 0.0001) and lesions detected qualitatively (*R*_pb_ = 0.32, *p* = 0.0016) demonstrated positive correlation between size and infarction. Seventy-three percent (*p* = 0.018) of lesions which demonstrated an increasing size from CBV, cerebral blood flow, to MTT/time to peak were true positive. Used in isolation, computer-generated lesion maps in CTP provide limited diagnostic utility in predicting infarct, due to their inherently low specificity. However, when used in conjunction with qualitative perfusion color map assessment, the lesion maps can help improve specificity.

## Introduction

CT perfusion (CTP), in conjunction with CT arteriography (CTA), and non-contrast head CT (NCCT) have been increasingly advocated for identifying appropriate patients for revascularization in the setting of acute stroke [1, 2] and are becoming more widely available in emergency settings [3, 4]. Previous studies focused primarily on the use of CTP for thrombolytic treatment assessment by evaluating for the existence of an ischemic penumbra [5, 6]. Only a few studies are available in the literature to assess the accuracy of CTP in diagnosing an acute infarct [7, 8], and none with quantitative lesion analysis for the purposes of diagnostic accuracy. Previous papers comparing CTP to diffusion-weighted imaging (DWI) only included evaluation of the core infarct and surrounding potentially salvageable ischemic penumbra in patients with known middle cerebral artery (MCA) infarcts or dense hemispheric stroke symptoms, leading to a study population including only acute stroke patients [9, 10]. To our knowledge, only one recent study in the literature assesses the practical use of CTP as a diagnostic study among patients who presented to the ED with stroke-like symptoms [4].

It has been shown that addition of CTP and CTA to NCCT does not adversely increase the time to tPA treatment for acute stroke patients in the ED [11]. However, before widespread utilization of this technique is feasible, further studies are needed in assessing the practical use of CTP with commercially available software as a diagnostic tool for patients presenting with stroke-like symptoms. Rapid assessment provided by simple post-processing of automated lesion maps generated from the software will help in timely management for potential thrombolytic therapy.

Our study will assess the diagnostic accuracy of CTP as a result of a single vendor commercially available software in predicting infarct by correlation with follow-up DWI. Individual lesion characteristics such as ischemia, infarct, or mixed (containing both infarct and ischemia) as well as lesion size will be assessed for correlation with DWI abnormality. The diagnostic accuracy of lesion maps generated by the software will be compared to a qualitative evaluation of the four major perfusion parameters: cerebral blood volume (CBV), cerebral blood flow (CBF), mean transit time (MTT), and time to peak (TTP).

## Materials and methods

This HIPAA-compliant study was approved by the Indiana University–Purdue University at Indianapolis Institutional Review Board with waiver of informed consent.

### Patient selection

At our institution, a primary stroke center, an acute stroke workup for patients presenting within 6 h of symptom onset consists of a NCCT followed by CTP and CTA of the head and neck. A retrospective analysis was performed to identify patients who had received an acute stroke workup and a follow-up MRI/DWI within 6 h of the initial CTP from August 2008 to August 2010. Because of a lack of a standardized protocol for MR imaging, the time between the initial CTP and follow-up MRI varied widely among patients. A 6-h interval was arbitrarily chosen for this study to decrease the chance of an interval development of acute infarction between exams while ensuring adequate sample size. Our exclusion criteria included patients who received intravascular thrombolysis between CTP and DWI, presence of intracranial arteriovenous shunting, and inadequate CTP due to technical difficulties (e.g. excessive motion, suboptimal bolus timing, and insufficient post-processing).

### CTP parameters

All studies were performed on a 64-slice CT scanner (Philips Brilliance 64, Philips Healthcare, Andover, MA). Eight contiguous slices at 5 mm thickness for a total of 40 mm of coverage were obtained with cine scanning for a total of 40 s. Iodinated non-ionic contrast material of 40 ml (Isovue-370; Bracco Diagnostics, Princeton, NJ) was injected via an arm vein at infusion rate of 4 ml/s. The majority of the MCA circulation centered at the basal ganglia was imaged. Post-processing was performed by trained technologists on Extend Brilliance Workstation CT perfusion package (Philips Healthcare, Cleveland, OH) with regions of interest placed in the anterior cerebral artery for arterial input function and the superior sagittal sinus for venous output function in addition to midline selection to obtain color maps of CBV, MTT, and TTP. CBF maps are generated by CBV divided by MTT. This software depends on the central volume principle and utilizes a closed form (non-iterative) deconvolution for calculation of MTT. Areas under the time density curves were used to calculate CBV. Determination of ischemic lesions (MTT > 7 s or 145 % of the normal contralateral side) and infarct (CBV < 2.0 ml/100 g) has been previously described in the literature [12]. Post-processing parameters and technical factors including region of interest placement, the resulting signal to time intensity curves, midline selection, and excessive patient motion were reviewed by a neuroradiologist to assess for a satisfactory CTP study. Computer-generated lesion maps were reconstructed at 5 mm slice thickness.

### CTP analysis

**Fig. 1:**
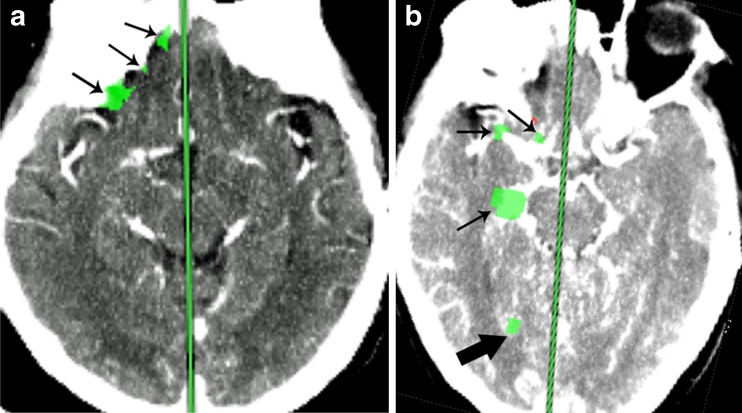
Examples of excluded lesions. **a** Lesions within areas of beam hardening (*small arrows*). **b** Lesions not projecting over the brain parenchyma. Vascular structures (*small arrows*) and choroid plexus (*large arrow*)

**Fig. 2:**
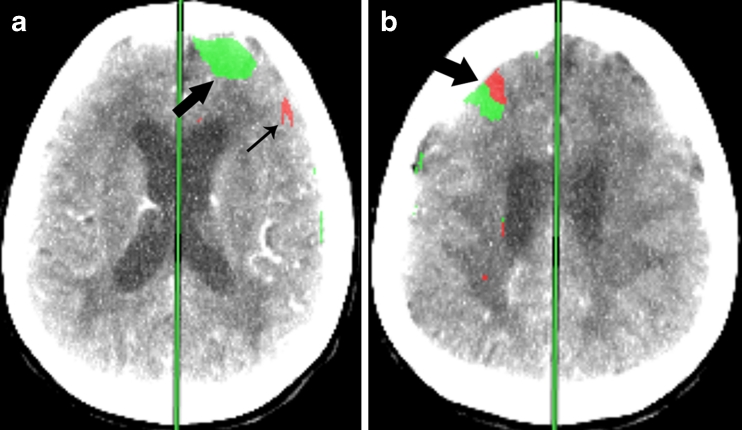
Lesion classification. **a** Ischemic lesions (*green*, *large arrow*) representing areas of elevated MTT and normal CBV. Infarct lesions (*red*, *small arrow*) representing areas of elevated MTT and reduced CBV. **b** Mixed lesions contained both areas of ischemia and infarct, the so-called “infarct core and ischemic penumbra” (*large arrow*)

**Fig. 3:**
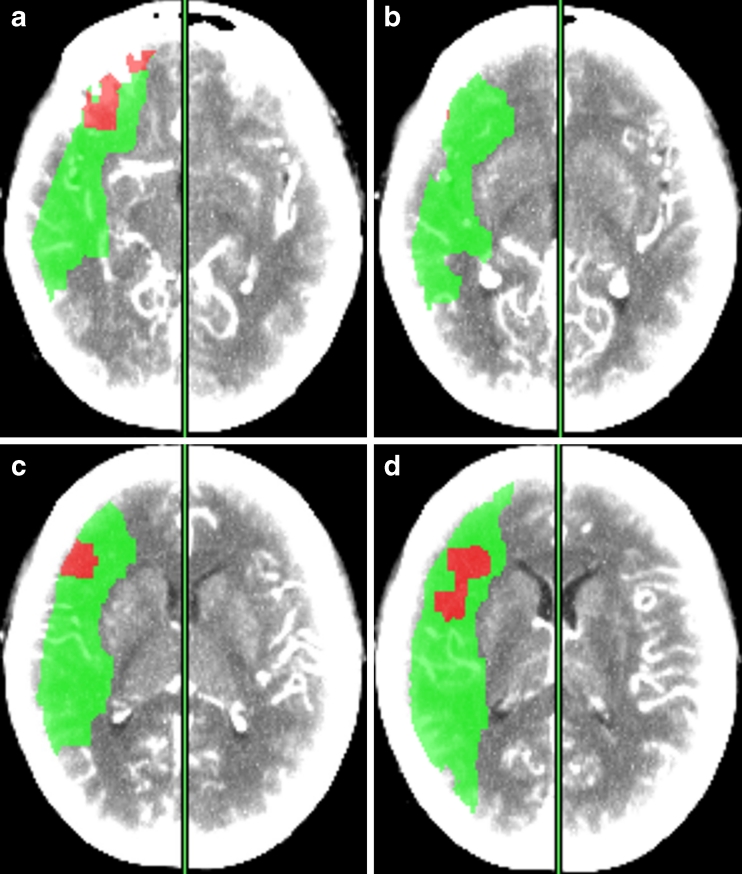
Four contiguous axial images demonstrating a large region of infarct core with ischemic penumbra. The areas of these contiguous lesions are summated into one larger lesion

Computer-generated lesion maps were evaluated for consensus by two neuroradiologists [7 years (CH) and 2 years (SH) of experience] blinded to color maps and DWI results. Analysis was performed on anatomic images with computer-generated superimposed colored lesions which met criteria for ischemia (elevated MTT with normal CBV, green) and infarct (elevated MTT with decreased CBV, red). Inclusion criteria for the computer-generated lesions were as follows: greater than 10 mm^2^ in area, lesions corresponding to brain parenchyma, and lesions not within areas of beam hardening adjacent to bone or chronic infarct on NCCT. All lesions not meeting these criteria were considered artifact and excluded (Fig. 1). Lesions were divided into three categories by perfusion parameters: ischemia, infarct, and mixed (containing both ischemic and infarct lesions) (Fig. 2). Lesion size was also measured by cross-sectional area. Contiguous lesions which persisted on the axial slice above or below another lesion and within the same vascular territory were summated into one larger lesion (Fig. 3). Volume was calculated by multiplying cross-sectional area by slice thickness to obtain milliliter volume units. CTP studies without any computer-generated lesions that met inclusion criteria were considered a negative study.

**Fig. 4:**
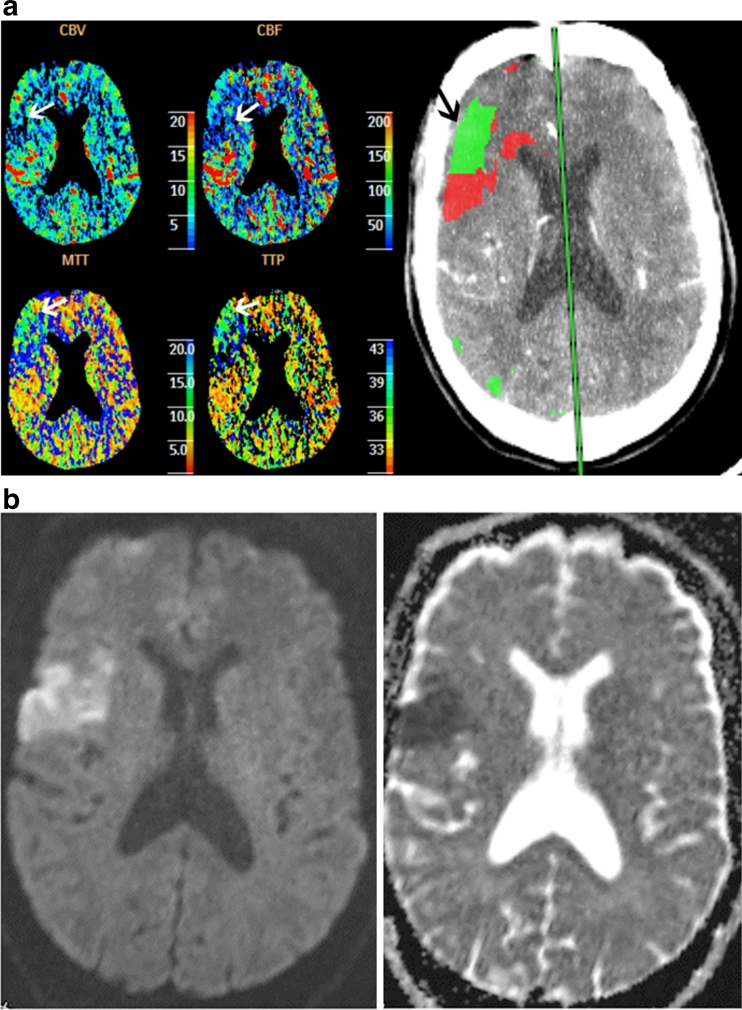
Sixty-five-year-old male presenting with slurred speech and facial droop. **a** Perfusion maps demonstrating a perfusion deficit (*open arrows*) with increasing areas from CBV to CBF to MTT/TTP. The corresponding computer-generated lesion map shows a mixed lesion (*closed arrow*). **b** MRI DWI and ADC map performed 4 h later demonstrate an area of decreased diffusion indicating acute infarct matching the area of decreased CBV on CT perfusion

Separate qualitative color map analysis of the four different parameters was performed at a later time blinded to both computer-generated lesion maps and DWI results. The four perfusion maps were displayed in a 16-scale color map. Observers were dependent on symmetry as well as a perceived change from the surrounding cortex (Fig. 4). When a perfusion deficit was suspected, correlation with source images were performed to exclude those caused by beam-hardening artifact or chronic infarct. If the perfusion deficit was included, the cross-sectional area of the lesion was measured and summated with contiguous perfusion deficits in the same vascular territory similar to the method as described for computer-generated lesions. Volume conversion was also calculated.

### DWI and statistical analysis

DWI and apparent diffusion coefficient (ADC) maps were assessed for areas of restricted diffusion, which were defined as areas hyperintense on DWI and hypointense on ADC map relative to adjacent normal brain parenchyma. Areas of restricted diffusion were then compared with the initial CTP study for anatomic correlation. To be considered as a true positive (TP) lesion for acute infarct on the CTP, it must have an anatomically corresponding area of restricted diffusion, regardless of the size of lesion on DWI. A false positive (FP) lesion had no corresponding restricted diffusion. A false negative (FN) lesion was a lesion with restricted diffusion on the DWI that was not identified on the initial CTP.

A true negative (TN) study was one that had no CTP lesions by inclusion criteria and no areas of restricted diffusion on DWI. Studies with areas of restricted diffusion outside of the corresponding imaged anatomy on CTP were included in the statistical calculations as FN studies. A TP study was any CTP study with a TP lesion, even if there were other coexisting FP lesions on the same study. CTP studies with both FP and FN lesions were counted as FP studies. This methodology was selected due to the increased likelihood of treatment with a positive result on CTP whether true or false.

Sensitivity, specificity, positive predictive value (PPV), and negative predictive value (NPV) were calculated for each CTP study as a whole. PPV for each category of lesion were also calculated. For each lesion, an *x* value of 1 was assigned for TP lesions and 0 for FP lesions. Point biserial correlation coefficient was calculated to determine the relationship between the size of the lesion and agreement with DWI.

For the qualitative color map analysis, the study as a whole was counted as TP if any of the four color maps demonstrated a perfusion deficit correlating with DWI, even if other FP or FN lesions were present on the same study. Again, infarcts on DWI not included in the scanned anatomy on CTP were counted as FN. For studies with both FP and FN areas, the study was counted as a FP. TP, FP, TN, and FN results were also assessed for each individual parameter of CBV, CBF, MTT, and TTP for each lesion, as a few studies were determined to have more than one lesion which were not in the same vascular territory. Point biserial correlation was also performed for lesion size for all color maps and separately for each of the four perfusion parameters.

Finally, the lesions perceived on the qualitative analysis of the color maps were compared to the computer-generated maps to assess whether a computer-generated lesion was present in the same anatomical region. These were then considered a negative area, and adjusted diagnostic accuracy was calculated for the study and for each of the four perfusion parameters.

## Results

A total of 73 patients were identified meeting inclusion criteria. Seven patients were excluded (three patients for receiving thrombolysis, four patients for inadequate CTP technique, and one patient for the presence of an intracranial arteriovenous malformation), and 65 patients (41 females and 24 males, age range 22–92 years, mean 57) were included in the study. Clinical presentation is summarized in the included table (Table 1). Twenty-two (34 %) of the 65 patients had acute infarcts on DWI: five patients had lacunar infarcts of the white matter and thalami, nine had focal infarcts in the MCA distribution with ASPECTS > 7, and eight patients had regional MCA distribution infarcts or larger ASPECTS ≤ 7. CTP to DWI interval was 0.6–6 h (mean 4.1 h).

**Table 1:** Clinical presentation of included patients presenting with stroke-like symptoms

Hemiparesis	29	Dysarthria	3
Numbness and confusion	10	Homonymous hemianopsia	1
Facial droop	9		
Aphasia	8		
Generalized weakness	5		

### Computer-generated lesion maps

There were 15 TP studies, 10 TN, 33 FP, and 3 FN. Four studies had both FP and FN lesions and were counted as FP studies for statistical analysis. Only one study had an area of restricted diffusion that was not anatomically included on the initial CTP study. This study also had FP lesions and was therefore counted as a FP study. Parameters of diagnostic accuracy are summarized within the following chart (Table 2).

**Table 2:** Diagnostic accuracy for computer-generated lesion maps, qualitative color map analysis alone, and qualitative analysis in conjunction with computer-generated lesion maps to exclude areas not detected on the latter

	Computer-generated lesion maps (%)	Qualitative analysis (%)	Qualitative analysis and computer lesion maps (%)
Study sensitivity	83 (0.95 CI 58–96)	70 (0.95 CI 46–87)	67 (0.95 CI 43–85)
Study specificity	21 (0.95 CI 11–36)	69 (0.95 CI 53–81)	80 (0.95 CI 64–90)
Negative predictive value	77 (0.95 CI 46–94)	84 (0.95 CI 67–93)	83 (0.95 CI 68–92)
Positive predictive value	29 (0.95 CI 18–43)	50 (0.95 CI 31–69)	61 (0.95 CI 39–80)

*CI* confidence interval

**Fig. 5:**
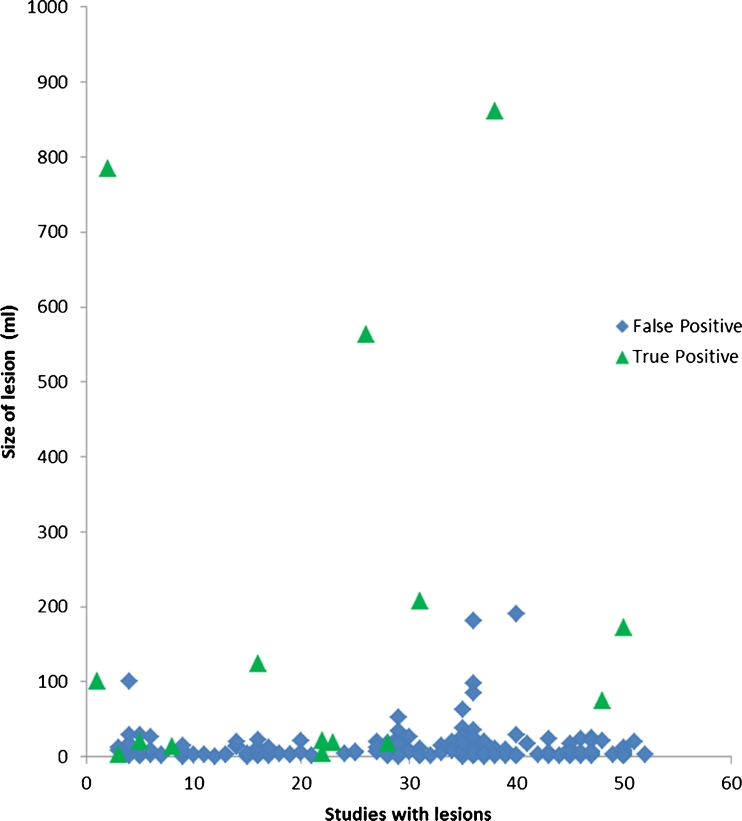
Scatter plot for all lesions meeting inclusion criteria on computer-generated lesion maps

Of the abnormal CTPs, there were 215 separate lesions (0.7–861 ml, mean 25 ml). Lesion categorization and PPV results are summarized below (Table 3). Point biserial coefficient was 0.46 (*p* < 0.0001), demonstrating a positive correlation of increasing size with the likelihood of a true positive lesion (Fig. 5, Table 5).

**Table 3:** Lesion categorization for computer-generated maps and positive predictive values, and 100 ml was chosen as an arbitrary cutoff value

Ischemic lesions	Lesions ≥ 100 ml
* N* = 143	* N* = 10
PPV = 6 %	PPV = 70 %
Infarct only lesions	Lesions ≤ 100 ml
* N* = 15	* N* = 205
PPV = 0 %	PPV = 6 %
Mixed (ischemic and infarct)	
* N* = 57	
PPV = 12 %

### Qualitative color map analysis

**Fig. 6:**
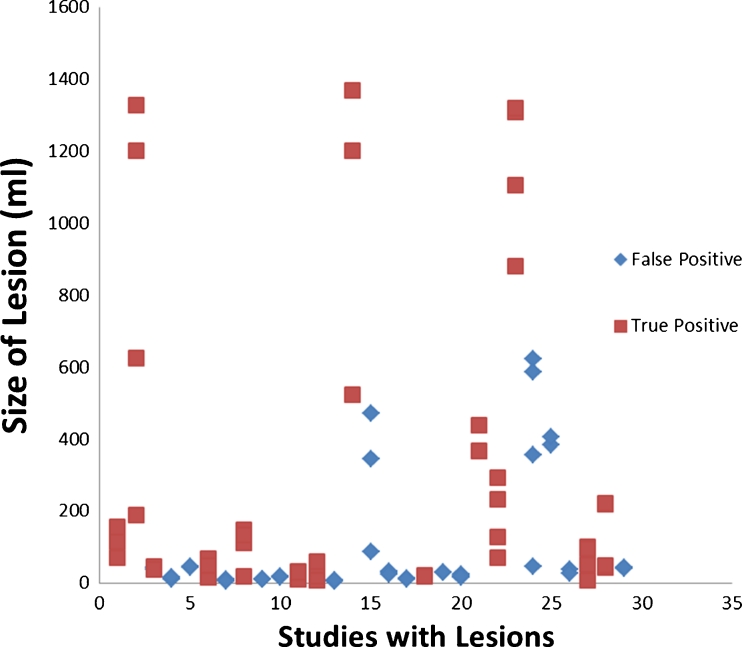
Scatter plot for all lesions from qualitative interpretation of color maps

For the study as a whole, there were 12 TP, 12 FP, 31 TN, and 6 FN studies. There were two studies with both FP and FN lesions which were counted as FP studies. There was one study with both TP and FN areas and another study with TP and FP areas; both were counted as TP studies. Parameters of diagnostic accuracy are summarized below (Table 2). There were five studies for which there were two noncontiguous lesions, resulting in *n* = 70 for the calculation of diagnostic accuracy for the four perfusion parameters. The results are summarized below (Table 4). Point biserial correlation demonstrated positive correlation of increasing size with increasing likelihood of a true positive lesion for all detected lesions as well as the four individual perfusion parameters; however, this was only statistically significant when calculated for all lesions regardless of the individual perfusion parameter (Table 5, Fig. 6). Interestingly, all detected lesions had at least MTT and TTP abnormality, which demonstrated similar cross-sectional areas for these two perfusion parameters. In those lesions with at least a CBF abnormality, the pattern of increasing lesion sizes from CBV, CBF, to MTT/TTP were associated with a PPV of 73 % (Fisher exact test *p* = 0.018) (*N* = 15, TP = 11, FP = 4). By contrast, all of the four lesions that did not have this increasing pattern were FP lesions. Lesions with only MTT/TTP abnormalities had a 27 % PPV (*N* = 11, TP = 3, FP = 8).

**Table 4:** Diagnostic accuracy for perfusion parameters for qualitative interpretation before and after excluding lesions not found on the computer-generated lesion maps

	Qualitative evaluation only (%)	Qualitative analysis and computer lesion maps (%)
CBV		
Study sensitivity	30 (0.95 CI 14–53)	30 (0.95 CI 14–53)
Study specificity	87 (0.95 CI 74–95)	91 (0.95 CI 79–97)
Negative predictive value	72 (0.95 CI 58–83)	73 (0.95 CI 60–83)
Positive predictive value	54 (0.95 CI 26–80)	64 (0.95 CI 32–88)
CBF		
Study sensitivity	48 (0.95 CI 27–69)	48 (0.95 CI 27–69)
Study specificity	83 (0.95 CI 69–92)	89 (0.95 CI 76–96)
Negative predictive value	76 (0.95 CI 62–87)	78 (0.95 CI 64–88)
Positive predictive value	58 (0.95 CI 34–79)	69 (0.95 CI 41–88)
MTT		
Study sensitivity	61 (0.95 CI 39–80)	61 (0.95 CI 39–80)
Study specificity	68 (0.95 CI 53–80)	77 (0.95 CI 62–87)
Negative predictive value	78 (0.95 CI 62–89)	80 (0.95 CI 65–90)
Positive predictive value	48 (0.95 CI 30–67)	56 (0.95 CI 35–75)
TTP		
Study sensitivity	61 (0.95 CI 39–80)	61 (0.95 CI 39–80)
Study specificity	66 (0.95 CI 51–79)	77 (0.95 CI 62–87)
Negative predictive value	78 (0.95 CI 61–89)	80 (0.95 CI 65–90)
Positive predictive value	47 (0.95 CI 29–65)	56 (0.95 CI 35–75)

**Table 5:** Mean and range of the cross-sectional area of true and false positive lesions with point biserial coefficients

	*N*	False positive mean (range), ml	True positive mean (range), ml	Point biserial coefficient (*R*_pb_)	Two-tailed *P*
Computer-generated lesions	215	12 (0.7–190)	160 (3.3–861)	0.46	<0.0001
All lesions (qualitative)	91	90 (4.0–623)	317 (6.4–1368)	0.32	0.0016
CBV	13	21 (6.3–47)	173 (6.4–880)	0.33	0.27
CBF	19	66 (5.6–356)	246 (9.4–1105)	0.31	0.19
MTT	29	109 (4.0–623)	390 (21–1368)	0.35	0.06
TTP	30	110 (5.6–587)	371 (18–1318)	0.35	0.06

Five lesions that were detected qualitatively on perfusion analysis were not detected by the computer-generated lesion map. None of these lesions were TP results. By weighing the negative results of the automated lesion maps over the positive qualitative interpretation and considering these studies as negative results, four became TN, and one, FN because this study failed to detect an area which was positive on the follow-up DWI. Recalculation of diagnostic accuracy of the study as a whole as well as for each of the four perfusion parameters after correcting FP to TN for these five lesions resulted in an increase in specificity and PPV (Tables 2 and 4).

Qualitative analysis had two FN results which were detected as TP lesions by the computer-generated lesion maps. All seven FN areas on the computer-generated lesion maps could not be detected by qualitative evaluation. Four of these had other FP lesions. By contrast, of the nine FN areas on qualitative analysis, only three had positive lesions elsewhere. Only one FN was outside of the covered anatomy. Of the eight FN areas which were covered by CTP, all were small lacunar or cortical infarcts with a mean volume of 7.7 ml (0.85–34.4).

## Discussion

Based on our study, the evaluation of computer-generated lesion maps from a single vendor for CTP for predicting acute infarct is a sensitive diagnostic tool but is limited by a lack of specificity and positive predictive value. Categorizing lesions based on perfusion parameters does not seem to increase the positive predictive value of the lesions. However, there is a significant positive correlation with large lesion sizes and infarct outcome. This corroborates the use of CTP as a tool for decision-making in treatment of infarcts with large ischemic penumbras.

By contrast, qualitative interpretation of the four different perfusion parameters (CBV, CBF, MTT, and TTP) improves diagnostic accuracy with respect to specificity and negative and positive predictive values but decreases the overall sensitivity. When computer-generated lesion maps are used to exclude perceived lesions from qualitative color map analysis, specificity and positive predictive value are further improved.

As previously described in the literature, CBV has the highest specificity of the perfusion parameters for infarct core but suffers from lower sensitivity, while MTT and TTP have higher sensitivities, but lower specificity [6, 7]. CBF is consistently in between CBV and MTT/TTP in diagnostic accuracy for both sensitivity and specificity [18]. Interestingly, 73 % of our lesions with increasing size in order from CBV, CBF, to MTT/TTP were true positive, suggesting that this pattern is helpful in diagnosing acute infarcts. It is also consistent with the hypothesis described previously that most acute infarcts have some region of ischemic penumbra larger than the infarcted tissue [13, 14].

Given the NPV of both the qualitative evaluation and computer-generated lesion maps, perhaps the most useful aspect of CTP as a diagnostic tool is as a screening exam to exclude stroke when the CTP is considered a negative study based on our criteria. DWI, although highly accurate, is not 100 % sensitive in diagnosing acute strokes [15]. Our use of DWI as the gold standard in our study reflects the common practice of using this modality as the imaging gold standard in confirming acute stroke. The overwhelming majority of the false positive lesions in our study was ischemic lesions, specifically, lesions that demonstrate elevated MTT and normal CBV. These lesions may not be truly false positive as areas of chronic hypoperfusion, and reversible ischemia may not always progress to infarct and, therefore, may not be detectable on DWI. This may explain our low specificity. A more accurate comparison of the ability of CTP to evaluate potentially reversible ischemic lesions would be to compare the technique to other perfusion studies such as dynamic susceptibility contrast or arterial spin labeling MR perfusion [16]. However, this is not within the scope of our study. Furthermore, comparing CTP to DWI represents a more accurate measure of risk for progression to acute infarct [17].

Our results contrast significantly with previous studies which reported high sensitivity and specificity for CTP in the diagnosis of acute stroke [4]. Study population may account for some of the discrepancy as previous studies included a higher percentage of stroke patients with larger volume strokes due to their inclusion criteria [10]. Our study included a cross-sectional representation of patients with stroke-like symptoms and subtypes presenting to the ED, including small lacunar infarcts, which contributed to the majority of our false negative results, and for which the treatment with thombolytics remain controversial [18].

Another factor contributing to discrepancies are possible technical differences between imaging parameters and post-processing algorithm. Recent literature demonstrates significant differences in CTP maps when using different commercially available software with different post-processing algorithms [19]. This underscores the necessity for standardization of CTP imaging parameters and software prior to practical mainstream utilization within the ED for both diagnosis and treatment of stroke [20].

Study limitations include the use of 6 h as the DWI time limit cutoff. It is possible that an interval infarct may occur within the 6 h of hospitalization, leading to a greater number of false negatives. However, with the false negative rate in our study (17 % for computer-generated maps and 33 % for qualitative analysis), this would only lead to modest gains in sensitivity and specificity. Newer 64 and 128 scanners have the capability of scanning the entire brain for CT perfusion, which may lead to less false negative studies [21]; however, only one infarct was outside of the CTP coverage in our study.

## Conclusion

Used in isolation, computer-generated lesion maps from a single vendor for CTP provide limited diagnostic utility in predicting infarct, due to their inherently low specificity. However, when used in conjunction with qualitative perfusion color map assessment, the lesion maps can help improve specificity. CTP characteristics which best correlate with true infarcts are a large lesion size and a pattern of increasing size from CBV, CBF, to MTT/TTP, confirming that most acute infarcts possess a larger ischemic penumbra.

## Abbreviations

CTP: CT perfusion
CTA: CT arteriography
NCCT: Non-contrast head CT
ED: Emergency department
CBV: Cerebral blood volume
CBF: Cerebral blood flow
MTT: Mean transit time
TTP: Time to peak
TP: True positive
FP: False positive
TN: True negative
FN: False negative
ASPECTS: Alberta Stroke Program Early CT Score
